# Effect of Supplementation on Levels of Homovanillic and Vanillylmandelic Acids in Children with Autism Spectrum Disorders

**DOI:** 10.3390/metabo12050423

**Published:** 2022-05-09

**Authors:** Paulina Gątarek, Joanna Kałużna-Czaplińska

**Affiliations:** 1 Faculty of Chemistry, Institute of General and Ecological Chemistry, Lodz University of Technology, 116 Zeromskiego Street, 90-924 Lodz, Poland; paulina.gatarek@dokt.p.lodz.pl; 2 CONEM Poland Chemistry and Nutrition Research Group, Lodz University of Technology, 90-924 Lodz, Poland

**Keywords:** autism spectrum disorders, ASD, supplementation, behaviour, chromatographic techniques

## Abstract

Autism Spectrum Disorders (ASD) are characterized by numerous comorbidities, including various metabolic and nutritional abnormalities. In many children with ASD, problems with proper nutrition can often lead to inadequate nutrient intake and some disturbances in metabolic profiles, which subsequently correlate with impaired neurobehavioural function. The purpose of this study was to investigate and compare the relationship between supplementation, levels of homovanillic acid (HVA) and vanillylmandelic acid (VMA) and the behaviour of children with ASD using quantitative urinary acid determination and questionnaires provided by parents/caregivers. The study was carried out on 129 children between 3 and 18 years of age. HVA and VMA were extracted and derivatized from urinary samples and simultaneously analyzed by gas chromatography-mass spectrometry (GC-MS). In addition, parents/caregivers of children with ASD were asked to complete questionnaires containing information about their diet and intake/non-intake of supplements. The application of the Mann–Whitney U test showed a statistically significant difference between the level of HVA and vitamin B supplementation (*p* = 1.64 × 10^−2^) and also omega-6 fatty acids supplementation and the levels of HVA (*p* = 1.50 × 10^−3^) and VMA (*p* = 2.50 × 10^−3^). In some children, a reduction in the severity of autistic symptoms (better response to own name or better reaction to change) was also observed. These results suggest that supplementation affects the levels of HVA and VMA and might also affect the children’s behaviour. Further research on these metabolites and the effects of supplementation on their levels, as well as the effects on the behaviour and physical symptoms among children with ASD is needed.

## 1. Introduction

Autism Spectrum Disorders (ASD) are neurodevelopmental disorders whose symptoms include communication disorders, abnormal interest and behaviour patterns and abnormal social interactions. The definition of ASD covers all types of autism, from childhood autism, through atypical autism, to Asperger’s syndrome and Kanner’s syndrome [1,2]. Autism is usually diagnosed around the age of three; however, it can often be diagnosed much later [3,4]. The prevalence of ASDs has increased. According to the Center for Disease Control and Prevention, on average 1 in 59 children is diagnosed with ASD [5]. Numerous studies have not identified a clear aetiology of autism. Recently, autism is considered a multicausal disorder, which indicates that there may be many reasons for its occurrence [6]. The aetiology of ASD is considered to be multifactorial, with both genetic and non-genetic (with the possibility of heritability being influenced by environmental factors that affect phenotypic expression) factors playing a role [6]. It is widely believed that ASDs are influenced by a variety of environmental factors, including neurotoxins, infections and maternal infections, but also genetic and immunological factors which, during critical periods, alter developmental processes by initiating some central nervous system (CNS) abnormalities and activating inflammatory processes in the nervous system. The following factors are also mentioned: parental age, maternal nutritional and metabolic status, infections during pregnancy, prenatal stress and exposure to heavy metals, toxins or drugs. The pathophysiology of autism is also influenced by mitochondrial dysfunction, methylation and trans-sulphuration cycle disturbances, abnormalities in the immune system and increased risk of oxidative stress. Particular attention should be paid to the maternal nutritional status throughout pregnancy, which is one of the most important elements for proper brain development. A deficit or an excess of micronutrients, including zinc, iron, folic acid, vitamin D and omega-3 fatty acids, may result in impaired neurodevelopment [7,8,9,10]. In recent years, increasing attention has been paid to the nutritional status of children with ASD. The complex psychopathological picture that includes restricted interests or repetitive behaviour, disability in social reciprocity and abnormal communication that are characteristic of ASD strongly influences children’s eating behaviour [11,12,13,14]. Epidemiological studies reported that 46–89% of children with ASD exhibit feeding difficulties (five times more common among children with ASD than typically developing children) [13]. Children with autism often eat selectively and limit their diet to a few products, often of low nutritional value. Partly for this reason, children may not have a sufficient number of vitamins, minerals or essential amino acids, which can cause an imbalance in the whole body. Chronic diarrhea, constipation or gastroenteritis can also be the reason why they do not get enough nutrients [11,12]. Nutrients are essential for the proper development of children. They are, among others, building blocks or cofactors of numerous enzymatic reactions such as the production of neurotransmitters [15]. Nutrient deficiencies which are observed in children with ASD include vitamins (C, D), omega-3 fatty acids and folic acid [16]. In addition to supplementation, various types of diets with some specific food products excluded, such as rice, citrus or eggs, are often used. Gluten-free, sugar-free, dairy-free, casein-free, antifungal and hypoallergenic diets are among the most commonly used diets for ASD. Although their influence on the improvement of the condition of children with autism has not been thoroughly researched, the observations of parents/caregivers indicate positive changes in the behaviour of some children [17].

A diagnosis of autism is currently established based on behaviour observations, standardized behavioural scales using the criteria of the fifth edition of the Diagnostic and Statistical Manual of Mental Disorders (DSM-5) and interviews with parents [18]. They provide information on potential biomarkers of autism and allow the creation of metabolic profiles that can be used to make individual treatment plans and to diagnose children early. The metabolic profile shows the dynamics of the biological system in response to genetic changes and provides information on changes caused by external stimuli (e.g., pharmacotherapy, diet) as well as on biological variability within the subpopulation (metabolic phenotype) [19]. Currently, metabolic profiling has become a very important tool in the diagnosis of many diseases, including cancer [20,21,22,23], metabolic syndrome [24], neurological disorders [25] and oxidative stress [26]. Combined methods are widely used in metabolomics studies, including gas/liquid chromatography coupled with mass spectrometry in various connection variants [27,28,29,30,31]. Chromatographic techniques allow quantitative analysis of nutrients and metabolites in body fluids and tissues, which in turn enables the determination of individual nutrient uptake and identification of biomarkers of metabolic disorders. Chromatographic techniques combined with mass spectrometry are used to evaluate major metabolic changes occurring in the body, and in combination with multivariate analysis have become a valuable strategy in metabolomics research [32]. Because of high separation efficiency and reproducibility of fragmentation patterns in electron ionization mass spectra, gas chromatography coupled to mass spectrometry (GC-MS) is the ‘gold standard’ and often the first-choice method for the determination of organic acids. Before the GC analysis of metabolites in body fluids, extraction and derivatization of the target compounds are required [33,34,35].

Numerous studies focusing on the determination of biomarkers in the body fluids of children with autism can be found in the literature. Chen et al. (2019) used metabolic profiles to assess major metabolic features of urine from 156 children with ASD and 64 children without ASD based on determined organic acids using GC-MS. On the basis of these studies, out of 76 organic acids present in urine, 20 potential ASD marker metabolites, of which 14 were organic acids, were selected [36]. Metabolic changes in low molecular weight metabolites in urine in the research conducted by Kałużna-Czaplińska et al. (2014) were presented. Urine metabolites were measured in 14 children with ASD and 10 children without ASD by GC-MS. Twenty-one endogenous compounds were identified on the metabolic profile, 14 of which were organic acids. In the group of children with ASD compared with children without ASD, higher levels of organic acids, such as α-hydroxybutyric, oxalic, β-hydroxybutyric, succinic, L-threonic, α-hydroxyglutaric, p-hydroxyphenylacetic, tartaric, ribonic, citric acid and m-hydroxybenzoic acid, were observed while levels of organic acids, such as sebacic, butanoic, phosphoric and propionic acid, were reduced, which may be indicative of impaired energy and lipid metabolism in children with ASD [37]. Evaluation of the metabolic profiles of urine in the Lebanese population with ASD by using the complementarity of analytical platforms, such as nuclear magnetic resonance (NMR 1D (1H), NMR 2D (1H-13C)) and liquid chromatography coupled to high-resolution mass spectrometry (LC-MS), were presented by Bitar et al. (2018). To improve sensitivity and specificity, they used these complementary multiplatform analytical approaches. Abnormal concentrations of tyrosine, 2-hydroxybutyrate, creatine and glutamate made it possible to distinguish individuals with ASD from controls. Changes in concentrations of 2-hydroxybutyrate and citrate were observed in the ASD group, which indicates the disturbance of propanoate and citrate [38]. Elevated levels of citrate, succinate and glycolate in urine samples of children with ASD were found by Emond et al. (2013) [39]. In the study conducted by Mavel et al. (2013), the levels of β-alanine, glycine, taurine and succinic acid were significantly increased in the urine of children with ASD compared to controls [40]. In another study conducted by Kałużna-Czaplińska (2011), differences between the levels of 8 organic acids in the urine of children with and without autism were presented. Significantly higher levels of 2-oxoglutaric acid, isocitric acid, citric acid, 4-hydroxybenzoic acid, 4-hydroxyphenyl acetic acid, hippuric acid, adipic acid and suberic acid were observed in the urine of children with ASD [41]. In a further study by Gątarek et al. (2020) the following compounds were determined in 120 children with ASD using GC-MS: benzoic acid, p-hydroxybenzoic acid, p-hydroxyphenyl acetic acid and hippuric acid. We also checked the influence of probiotic supplementation on the level of carboxylic acids. We found statistically significant differences in supplementation with probiotics and the level of p-hydroxyphenyl acetic acid, which may suggest disturbances in the intestinal flora in children with ASD [42]. Kałużna-Czaplińska et al. (2010) found that levels of HVA and VMA were significantly increased in the urine samples of children with ASD (HVA 28.8 ± 15.5 μmol/mmol creatinine and VMA 22.2 ± 13.0 μmol/mmol creatinine) compared with controls (for HVA 4.6 ± 0.7 μmol/mmol creatinine and VMA 3.8 ± 0.6 μmol/mmol creatinine) [43]. Piras et al. (2022) examined the urine metabolome of children with ASD and their healthy siblings by proton nuclear magnetic resonance (1H-NMR) spectroscopy. Significant differences between them were observed. The levels of 2-hydroxybutyrate and hippurate were increased in children with ASD, while the level of isocitrate was decreased. The increase in 2-hydroxybutyrate was linked to oxidative stress, as well as to increased energy requirements in children with ASD. Furthermore, differences in the levels of the determined compounds may suggest perturbations in the pathways involved in the metabolism of asparagine and tyrosine [44].

An important source of information about metabolism and potential pathophysiological alterations in children with ASD is metabolites, such as organic acids, in particular, neurotransmitter metabolites; they provide information about the processes taking place in the human body. Organic acids excreted in the urine are the final or intermediate products of the metabolism of amino acids, sugars, biogenic amines, steroids, lipids and other endogenous compounds, but they can also come from external sources such as food additives or drugs [45]. The analysis of the levels of organic acid in the body fluids of children with autism provides important information on the following processes: fatty acid oxidation, carbohydrate metabolism, energy production, oxidative damage, intestinal dysbiosis, detoxification or disturbances in neurotransmitter metabolism [39,45,46,47]. The levels of some urine organic acids in children with ASD were often correlated with the impairment in the neurobehavioural function (manual dexterity, attention/response speed, visual perception, memory, perceptual motor speed and motor speed/steadiness [48]. Organic acids linked to neurobehavioural impairments are vanillylmandelic and homovanillic acids. Vanillylmandelic acid (4-hydroxy-3-methoximandelic acid, VMA) is the major final metabolite of adrenaline and noradrenaline in humans. Homovanillic acid (4-hydroxy-3-methoxyphenylacetic acid, HVA) is the last metabolite of the neurotransmitter dopamine in the human body, which is very similar to the structure of VMA. They are the main metabolites of catecholamines. The analysis of the levels of VMA and HVA in body fluids is used not only to diagnose many health problems but also to monitor the progress of treatment [49]. The simultaneous detection of HVA and VMA in urine may not only reflect the excretion of catecholamines in vivo, but it is also important for the early diagnosis and identification of central nervous system diseases [50].

In this study, we measured the levels of HVA and VMA in urine samples from children with ASD with and without supplementation. The aims of this study were to investigate and compare the relationships between supplementation, the levels of HVA and VMA concentrations, and the behaviour of children with ASD based on parents’ views expressed through the questionnaire about the nutrition and supplement intake of the children participating in the study. Additionally, based on preliminary questionnaire results, the study on determining whether oral vitamin supplementation significantly affects the concentration levels of selected metabolites was performed.

## 2. Results

The GC-MS method was applied determine the levels of homovanillic and vanillylmandelic acids in the urine samples collected from 129 children with autism from Poland. The results were calculated as a ratio of the HVA/VMA and urinary creatinine concentration and expressed in μmol/mmol of creatinine. The study group was divided into two subgroups, supplemented children with ASD and children with ASD without supplementation. In this study, we evaluated the influence of vitamin B (B1, B3, B6 and B12), vitamin C, vitamin D3, probiotics and omega-3 and omega-6 fatty acids supplementation on the levels of HVA and VMA in the urine of children with ASD. The supplementation table of observed levels of HVA and VMA and creatinine in the urine of children with autism are presented in Table S1 in Supplementary Materials. The levels of carboxylic acids in the case of supplemented children with ASD were 26.62 ± 12.54 μmol/mmol creatinine for HVA, 12.92 ± 8.32 μmol/mmol creatinine for VMA. The levels of HVA and VMA in the case of not supplemented children with ASD were 30.04 ± 13.32 μmol/mmol creatinine and 11.25 ± 4.15 μmol/mmol creatinine, respectively. The normality of the distribution of the variables was verified using the Shapiro–Wilk test (*p* < 0.05). No normal distribution was found for HVA (*p* = 7.99 × 10^−8^) and VMA (*p* = 2.70 × 10^−9^), respectively (Table 1). Individual differences in the levels of HVA and VMA between the two groups (supplemented children and children without supplementation) were found after performing the Mann–Whitney U test. The differences with a *p*-value lower than 0.05 were considered statistically significant. Determined levels of the metabolites and the supplementation with the specific supplements (vitamin B, vitamin C, vitamin D3 and omega-3 and -6 fatty acids) were considered, as shown in Table 2. The application of the Mann–Whitney U test showed a statistically significant difference in the level of HVA in the group of children with supplemented/non-supplemented vitamin B (*p* = 1.64 × 10^−2^), and for HVA and VMA in the group of children supplemented/not supplemented with omega-6 fatty acids *p* = 1.50 × 10^−3^ and *p* = 2.50 × 10^−3^, respectively. It did not show, however, any statistically significant difference between the level of VMA and vitamin B supplementation (*p* = 0.3620). No statistically significant differences were found between the levels of HVA and VMA and other supplementations, such as vitamin C supplementation (HVA *p* = 0.8679; VMA *p* = 0.3876), vitamin D3 supplementation (HVA *p* = 0.9823; VMA *p* = 0.1638), omega-3 fatty acids (HVA *p* = 0.4189; VMA *p* = 0.8189) and probiotics supplementation (HVA *p* = 0.1376; VMA *p* = 0.3962). Statistically significant differences can also be observed when considering the levels of determined metabolites and the effect of supplementation with B vitamins (B1, B3, B6 and B12). The differences with a *p*-value lower than 0.05 can be observed for HVA levels and vitamin B6 supplementation (*p* = 4.99 × 10^−2^) and for VMA with B3 (*p* = 1.06 × 10^−2^) and B12 (*p* = 3.71 × 10^−2^) supplementation, as shown in Table S2 and Figure S1 in the Supplementary Materials.

A significant number of children with ASD participated in the study. Children were divided in terms of taking/not taking supplementation. Due to the lack of a control group, the obtained results were compared with the reference values for the typical development of children (Table 1). Reference values in the urine were taken from the database available online at The Human Metabolome Database [51]. Based on this, it was found that HVA levels exceeded the reference levels almost 3 times and VMA almost twice. Moreover, in the previous study [43], for 36 neurologically normal children, mean levels of HVA and VMA 4.6 ± 0.7 μmol/mmol creatinine and 3.8 ± 0.6 μmol/mmol creatinine were obtained, respectively. The resulting metabolite levels for children with ASD compared to the values for normally developing children are significantly higher, five times and six times higher for HVA and VMA, respectively. None of the normally developing children received medications or any supplements.

Supplemented children with ASD were found to have lower mean and median urine levels of HVA and VMA compared to the children with ASD without supplementation. Based on the median difference, the average difference in the levels of urinary carboxylic acids between studied groups for HVA and VMA was 3.31 μmol/mmol creatinine, 0.68 μmol/mmol creatinine, respectively.

The application of the Mann–Whitney U test showed differences between age and the levels of HVA (*p* = 0.0021) and VMA (*p* = 0.0386), and BMI and the levels of HVA (*p* = 0.0293) and VMA (*p* = 0.0453). The gender of the patients does not seem to influence the concentration of either compound (*p* = 0.2658 for HVA and *p* = 0.1283 for VMA), as can be seen in Figure S2 in the Supplementary Materials. The results were obtained after the analysis of samples of children supplemented with vitamin B for the levels of HVA (*p* = 0.0164) and supplemented with omega-6 fatty acids for the levels of HVA (*p* = 0.0015) and VMA (*p* = 0.0025) were statistically significant.

**Figure 1:**
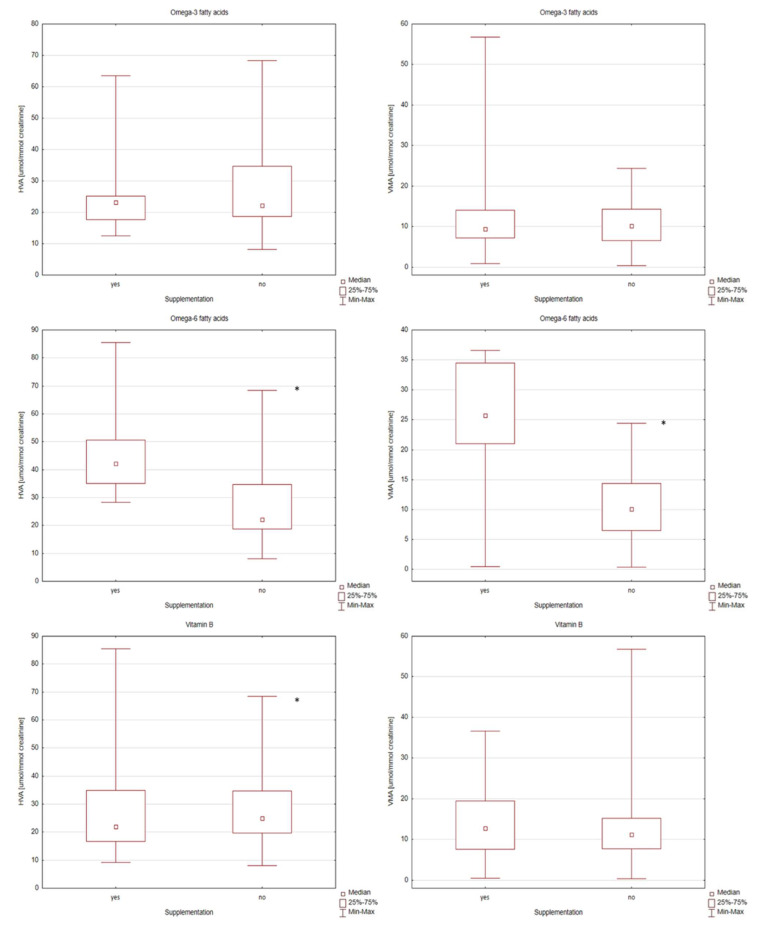
Box and Whisker plots for compounds determined in supplementation-categorized children with autism group (omega-3 and omega-6 fatty acids, vitamins B). In these box plots, medians inside the 25–75% interquartile range (IQR) are presented. *—statistically significant with *p*-value < 0.05.

**Figure 2:**
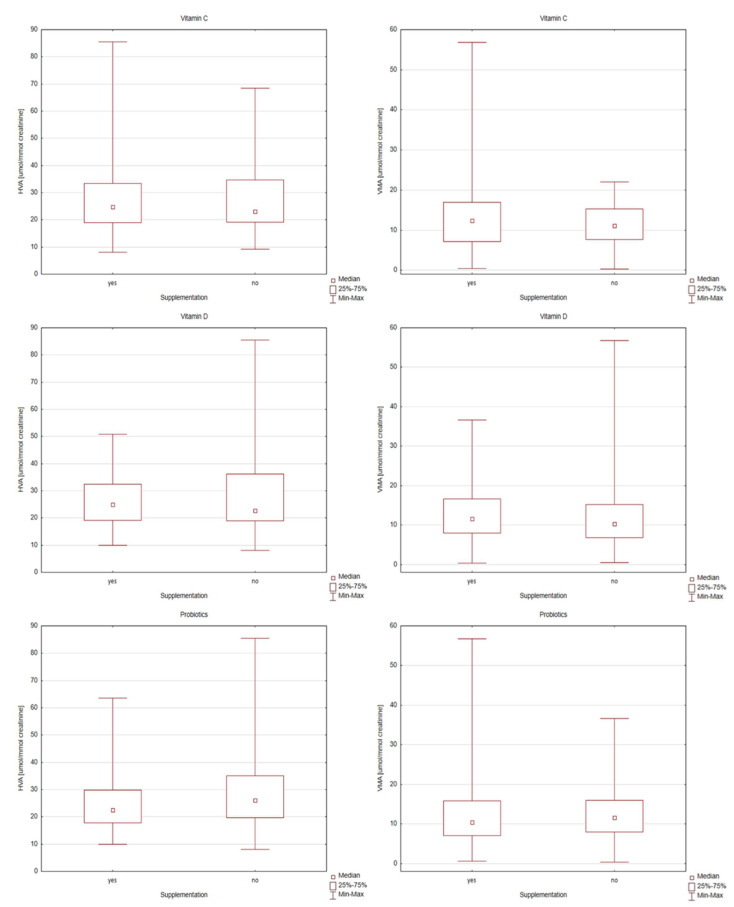
Box and Whisker plots for compounds determined in supplementation-categorized children with autism group (vitamin C, vitamin D and probiotic). In these box plots, medians inside the 25–75% interquartile range (IQR) are presented.

Individual differences in the levels of HVA and VMA in the supplementation-categorized ASD group were presented using box and whisker plots (Figure 1 and Figure 2). Box and Whisker plots allow simultaneous evaluation of the differences and variation of the results in the content of compounds determined by the study group. In the results of urine analysis of children with ASD supplemented with omega-3 fatty acids, the variability of the results of the level of VMA concentration was characterized by higher variability than those without supplementation (Figure 1). Evaluation of the effect of omega-3 supplementation on HVA levels indicated high variability of results in this group. Higher variability in the results in the omega-6 supplemented group of children regarding HVA and VMA levels was also observed. In the results of children with ASD supplemented with vitamin B, the variability of the results of the level of HVA concentration was higher than in those who did not receive supplementation. Contrary to the variability of the level of VMA in the samples of children with ASD supplemented with vitamin B, it was smaller in the case of children without supplementation. Figure 2 shows the results of the levels of HVA and VMA concentrations for the samples of children with ASD supplemented with vitamin C, vitamin D and probiotics. Taking into account the effect of supplementation with vitamins C and D and probiotics, no statistically significant changes in the levels of HVA and VMA concentrations were observed. Higher variability in the results on HVA was observed in the vitamin C supplemented group of children. Contrary to the variability of the level of HVA in the samples of children with ASD supplemented with vitamin D, it was smaller in the case of supplemented children (Figure 2). Due to the lack of relationship and correlation between the levels of HVA and VMA in the urine of children with autism and probiotic supplementation, this will not be discussed further in this manuscript.

Finally, the Spearman’s Rank Correlation Coefficient was used to verify if the results correlate to the supplementation (Table S3 Supplementary Materials). Nonparametric correlation analysis showed a strong positive correlation between the concentration of HVA and VMA (correlation value = 0.74, *p*-value = 1.50 × 10^−22^), and a weaker correlation between omega-3 and -6 fatty acids supplementation and concentration of HVA (correlation value −0.21, *p*-value = 0.0243) and a positive correlation between the level of VMA concentration and omega-3 fatty acids supplementation (correlation value 0.21, *p*-value = 1.55 × 10^−2^). In the study group, we also observed negative correlations between supplementation of omega-3 and -6 fatty acids and vitamin D (correlation value = −0.40, *p*-value = 3.13 × 10^−6^), and positive correlations between supplementation omega-3 and -6 fatty acids and vitamin B (correlation value = 0.35, *p*-value = 3.70 × 10^−5^), vitamin C (correlation value = 0.25, *p*-value = 3.89 × 10^−3^), and a weaker correlation between supplementation of vitamins C and D (correlation value 0.27, *p*-value = 1.65 × 10^−3^), and vitamins C and B (correlation value 0.37, *p*-value = 1.31 × 10^−5^).

**Figure 3:**
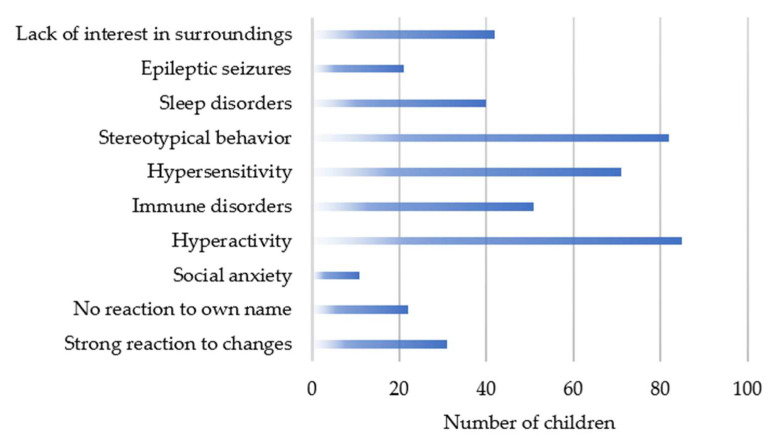
Observed types of behaviour in the study group of children with ASD.

In this study, we additionally present preliminary results from questionnaires completed by the parents/caregivers of the children with ASD. The results presented should be interpreted with particular caution due to the subjective assessment of children’s behaviour by parents. Parents/caregivers provided information on the weight, height, gender, age, consumed food and diets, food exclusion, supplementation and its dosage and duration and medications taken. Parents/caregivers were also asked in the questionnaires to evaluate their child’s behaviour. Presented results are based on observations made by parents/caregivers of children with autism. Based on the information provided by parents/caregivers in questionnaires on children’s behaviour, it was found that the most frequently observed behaviour of children with autism was hyperactivity (66%) and stereotypical behaviour (64%). Parents/caregivers of 55% of children reported hypersensitivity and only 9% reported social anxiety in their child. Data on the type of behaviour and the number of children in whom it was observed are presented in Figure 3.

In more than half of the cases, with the increase in the level of HVA, hyperactivity, stereotypical behaviour and immunity disorders increased. In more than half of the cases, with the increasing level of VMA, hyperactivity and stereotypical behaviour increased. Behaviours, such as social anxiety, epileptic seizures, not responding to one’s name, strong reaction to changes, sleep disorders and also immune disorders were not observed in over half of them.

## 3. Discussion

Recently, there has been an increased interest in finding metabolic pathways that are disrupted in autism. Researchers indicate that vitamin and mineral supplementation may affect certain physiological processes associated with metabolic and nutritional abnormalities in individuals with ASD, including methylation, sulfation, glutathione redox imbalances, mitochondrial dysfunction and oxidative stress. Moreover, nutritional status is indicated as a possible causal factor for attention and communication disorders. Increasing attention is being paid to the role of urinary HVA and VMA in the diagnosis and treatment of children with autism, but also, they are connected with neurotransmitter metabolism, and they were used in the diagnosis of some neurologic disorders [18,43].

Dopamine is a precursor of adrenaline and norepinephrine, however, urine studies of children with autism have shown that in participants with ASD, dopamine is not biotransformed into noradrenaline, but transiently into HVA, which was confirmed by elevated levels of this organic acid and a reduced level of VMA, which is the last metabolite of noradrenaline degradation [18]. Data analysis showed a higher level of homovanillic acid in the urine of children with ASD, both supplemented and not supplemented compared to the reference values. Elevated HVA levels may result from the reduced dopamine β-hydroxylase enzyme activity. The dopamine β-hydroxylase enzyme is a very important enzyme in catecholamine metabolism and converts dopamine to norepinephrine in noradrenergic neurons, adrenergic neurons and also adrenal chromaffin cells. Therefore, it controls the synthesis of norepinephrine as well as the ratio of dopamine to norepinephrine. The block of dopamine β-hydroxylase enzyme results in the accumulation of dopamine and therefore HVA, but also results in a lower production of noradrenaline and adrenaline, and lower first noradrenaline metabolite, 3-methoxy-4-hydroxyphenethyleneglycol (MHPG) as well as VMA, which is the last metabolite of noradrenaline degradation [52]. Dopamine is the essential modulator of neuronal activity in brain regions associated with ASD. For this reason, dysfunction of dopamine signalling may be associated with brain activity in the brain structure associated with ASD [53]. The high dopamine level is connected with severe deficits and related symptoms in ASD, such as motor dysfunction, stereotyped behaviours, convulsions and neurogenesis [18]. Changes in HVA levels in children with ASD may suggest some dopaminergic system disturbance, including mood disorders, disorders of social relationships, aggression and also repeated behaviours [43]. In our study, we also observed that increased HVA levels were associated with increased symptoms, including stereotyped behaviours, hyperactivity and also immunity disorders. Moreover, in children with ASD dopamine is converted directly into HVA, which was found to be significantly elevated in this study. Interestingly, higher HVA levels were observed in children taking supplementation, but not in the case of children with ASD who were not supplemented. In the literature, a relationship between the severity of ASD symptoms and HVA levels has been observed. The association between urinary HVA levels and increased stereotypical behaviour, agitation and reduced spontaneous behaviour has been reported. In the previous study conducted by Kałużna-Czaplińska et. al. (2010), the levels of HVA and VMA in comparison with the results from children from the control group were significantly higher in the urine of children with ASD [43]. In comparison with this study, the levels of HVA were similar to those from 2010, but VMA levels are lower in this research. The level of HVA excreted in the urine of children with ASD is not unequivocal. Some studies reported no difference between HVA and VMA levels in ASD participants compared to a normally developing control group [39,54,55]. Another study has reported increased levels of HVA in children with ASD [43], while in the study conducted by Martineau et al. (1992), the levels of HVA in urine decreased significantly with the age of the child [56]. A significantly higher HVA/VMA ratio is presented in the research conducted by Shaw (2010). This higher ratio may indicate a serious imbalance in epinephrine/norepinephrine and dopamine production. In contrast, high levels of the dopamine metabolite, HVA, indicates that oxidative stress caused by superoxide free radicals affects the brain, adrenal glands and sympathetic nervous system. Oxidative stress may result from untreated Clostridia bacteria [57].

Several approaches are used to treat and manage ASD. Among them, vitamin B6 is widely used to manage the symptoms observed in children with ASD. Vitamin B plays a critical role in methyl group donation for the synthesis of nucleic acids, lipids, proteins, hormones and neurotransmitters [11]. Deficiencies of vitamin B can lead to disorders of the nervous system, heart disease, deterioration of well-being, increased risk of behavioural and mood disorders, as well as disorders of cholesterol and homocysteine levels in body fluids. An excess of these vitamins is excreted in sweat, urine and faeces [58]. Vitamin B6 and magnesium are compounds involved in the synthesis of neurotransmitters, such as dopamine and serotonin, the abnormal levels of which have been observed in children with ASD. Vitamin B6 reduces ASD symptoms and reduces urinary excretion of HVA [59,60,61]. In the urine samples of children with autism who were supplemented with vitamin B6, a reduced concentration of HVA was observed [62]. On the other hand, in more recent studies, after supplementation with a high dose of vitamin B6/Mg, no adverse events were recorded, and it was concluded that this supplementation was not recommended, but at the same time, researchers recorded a significant reduction of the HVA level in urine [16]. Improved social interactions, communication and stereotypical behaviour were observed in children who were supplemented with vitamin B6 and magnesium [63]. However, studies on supplementation with methyl B12, which is a key co-factor in MET transmethylation/trans-sulphuration metabolism, have drawn the opposite conclusions. After B12 supplementation, clinically significant improvement in symptoms that are measured by the Clinical Global Impression scale was recorded [16]. Based on the 2016 study, children who were injected with methyl B12 made a significant improvement in their Clinical Global Impressions-Improvement (CGI-I) score compared to the group of children who received a placebo. However, on the Social Responsiveness Scale or the Aberrant Behaviour Checklist (ABC), which is rated by parents, no improvements were registered [11]. In our study, we observed a statistically significant difference between HVA in the group of children with ASD and vitamin B supplementation. Supplementation with vitamins of group B also reduced urinary HVA levels. Moreover, this supplementation also caused an increase in VMA levels.

Vitamin D is responsible for calcium and phosphate metabolism, as well as for maintaining the proper level of calcium in the blood. Deficiencies of this vitamin can lead to rickets in children and osteoporosis in adults. Overdose o vitamin D can lead to irreversible calcification of soft organs and tissues, hypertension, weakness and headaches [15]. According to research, when children with autism were supplemented with a high dose of vitamin D3, the clinical behaviour of children with ASD significantly improved according to behaviour scales such as the Aberrant Behaviour Checklist (ABC). However, in another research where children were supplemented with a smaller dose of vitamin D3, the results showed improvement only in self-care (according to Developmental Disabilities-Children’s Global Assessment Scale) [16]. In 2015, studies were conducted on children who were given a high dose of vitamin D for 3 months. Out of the 83 children who were receiving vitamin D during this time, 67 made an improvement in symptoms, such as attention, concentration, stereotyping, behaviour and eye contact, which are assessed using the Childhood Autism Rating Scale (CARS) and autism behaviour checklist. Subsequent studies showed that after the trial in which children were injected and administered vitamin D3 orally, the results on the CARS scale and on the autism checklist decreased significantly. It was also noted that the results of this supplementation were more noticeable in younger children [11]. In our research, the levels of HVA in the samples of children that did not get any vitamin D supplementation were higher than in those who were supplemented. VMA levels were similar in both groups.

Vitamin C facilitates the assimilation of iron and influences the synthesis of corticosteroids and some neurotransmitters. Deficiencies of this vitamin can cause brittleness of capillaries, skin changes, gum swelling, as well as tooth loss and bone fractures. Overdose of vitamin C is harmless, with excess vitamin C being excreted in urine [64]. Studies show that supplementation with vitamin C may positively influence the pathological behaviour of children with autism [65]. Other studies observed that the use of vitamin C supplementation reduced the severity of sensory-motor behaviours such as whirling, pacing and rocking [11,66]. Our research levels of HVA were higher in the samples of children who were not supplemented with vitamin C than those who took this supplementation.

Omega-3 and -6 fatty acids play complementary roles in neuronal structure and function. In our research, the level of VMA was higher in children who received omega-3 fatty acids supplementation than in children who were not supplemented. Levels of HVA are similar in subjects who received omega-3 fatty acids supplementation and those who did not receive it. We also observed a correlation between HVA and VMA levels in the group of children with ASD and omega-6 fatty acids supplementation. Supplementation with omega-3 and -6 fatty acids showed a positive correlation with the levels of HVA and VMA in the study group, and supplementation with omega-3 fatty acids and VMA levels. Because omega-3 fatty acids are found in cell membranes, they are important for the structure of the brain and its functioning, while omega-6 fatty acids are connected with ion currents, protein kinase activity and the induction of long-term potentiation. This process is associated with the consolidation of memory and learning. Supplementation with omega-3 fatty acids in combination with omega-6 fatty acids may be more beneficial to cognitive function than supplementation with omega-3 fatty acids only [67]. Introducing omega-6 fatty acid in the form of γ-linolenic acid (GLA) into supplementation may improve the anti-inflammatory effect of supplementation, as well as behavioural outcomes. The decreased concentration of omega-3 fatty acids was registered in children with ASD. Supplementation with omega-3 fatty acids, according to the research, causes improvement in reducing hyperactivity and stereotypes of children with autism, in comparison with the group which took a placebo. However, the results of analyses for subscales of the Aberrant Behaviour Checklist did not show significant differences between the two groups. In another study, after omega-3 fatty acids supplementation of children with autism, there was observed improvement in lethargy and stereotypy in ABC subscales, but on the other hand, differences in measures, such as CGI-I, were not registered [16,68,69]. Supplementation with omega-3 fatty acids reduces aggression [70], impulsiveness [71], hyperactivity and repetitive behaviours [72] while improving cognitive and motor skills, concentration and eye contact.

We identified a few study limitations that pose a challenge to our findings. Firstly, the lack of a control group consisting of age- and gender-matched normally developing children. Secondly, the study group of children with ASD was unequal in terms of gender. In addition, our analysis may have higher variability, which can have the effect of reducing the statistical power to detect associations. Thirdly, our preliminary results should be interpreted with caution as the questionnaires on a particular child’s behaviour were completed by parents/caregivers. Additionally, some of the questionnaires we received were complete. Fourthly, children with ASD participating in this study received supplements produced by different manufacturers. Fifthly, due to the fact that autism is a complex and multifactorial disease, it may require a larger sample size in order to capture more relationships and correlations between the factors under study. Lastly, our analysis may have been influenced by random factors an1d may be disturbed by unmeasured factors.

## 4. Materials and Methods

### 4.1. Subjects

**Figure 4:**
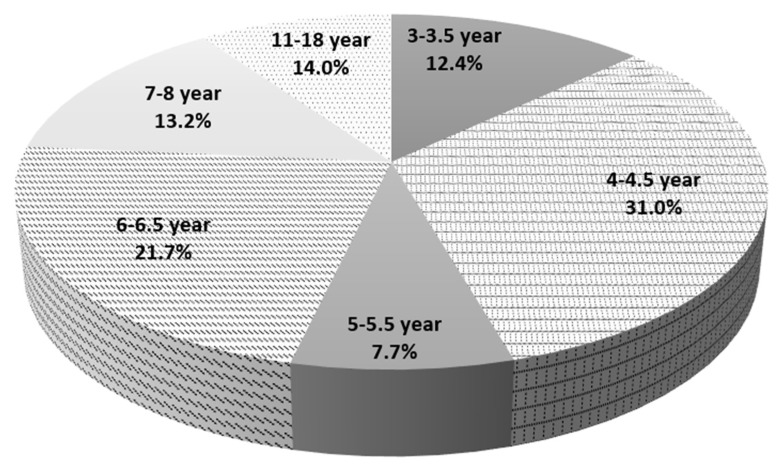
Age characteristics of study population.

129 subjects participated in the study (120 boys and 9 girls). The age range of the study participants was 3–18 years. Age characteristics of the study population are presented in Figure 4.

Psychiatry specialists and psychologists in the Center for Diagnosis and Therapy of Autism in Lodz Navicula (Poland) performed the ASD diagnosis according to the Diagnostic and Statistical Manual of Mental Disorders (DSM)-5 [73] criteria and confirmed it by using a Childhood Autism Rating Scale (CARS). Samples of morning urine were stored at −20 °C until analysis. The research was carried out with the approval of the Ethics Committee of the Polish Mother’s Memorial Hospital Research Institute on 30/2018.

The standard for monitoring a child’s growth and development is the measurement of length/height and body weight. Percentile charts for body height, weight and body mass index (BMI) in relation to age and weight in relation to body height for children and adolescents aged 0–18 years are available in Poland. A comprehensive study of the developmental norms of children and adolescents aged 0–18 years was created based on the current WHO International Growth Standards (for ages 0–3 years) [74] and the reports of the OLAF and OLA studies (for 3–18 years, n = 22,211 participants) [75,76]. The limits of underweight, overweight and obesity were developed separately for girls and boys according to the methodology presented by Cole and colleagues [77,78]. Based on the BMI and height of children and adolescents, using percentile charts, participants were classified into 4 groups: underweight (10.1%), normal weight (72.1%), overweight (17.1%) and obese (0.7%) (Table 3).

Before the urine analysis, parents and caregivers of the children were asked to fill in the questionnaire concerning information on the weight, gender, age, consumed food and diets, food exclusion, supplement dosage and duration, medications taken and the behaviour of the children. Information on the diet from the questionnaires showed that 38 children were on a casein-free diet, 26 on a gluten-free diet, a total of 26 on a sugar-free or sugar-reduced diet, 6 children on a low-carbonate diet, 7 children did not eat specific food products, such as rice, citrus, eggs, yeast or wheat, 9 children did not eat dairy products or lactose-containing products, and 59 children did not have any dietary restrictions.

The study involved 129 children with ASD, 104 children were supplemented while those without any supplementation were 25. Supplemented children with ASD received the following vitamins C (ascorbic acid), D3 (cholecalciferol), and vitamins B, such as B1 (thiamin), B3 (niacin), B6 (pyridoxine) and B12 (methylcobalamin). They also received omega-3 fatty acids: EPA (eicosapentaenoic acid) and DHA (docosahexaenoic acid), and also omega-6 fatty acids: GLA (γ-linolenic acid) and LA (linoleic acid). Supplemented children also received probiotics consisting of Lactobacillus (L. plantarum, L. rhamnosus L. acidophilus, L. lactis, L. paracasei, L. salivarius), Streptococcus salivarius, Bifidobacterium and Lactococcus bacteria. Specific characteristics of the studied population regarding supplementation are presented in Table 4.

**Figure 5:**
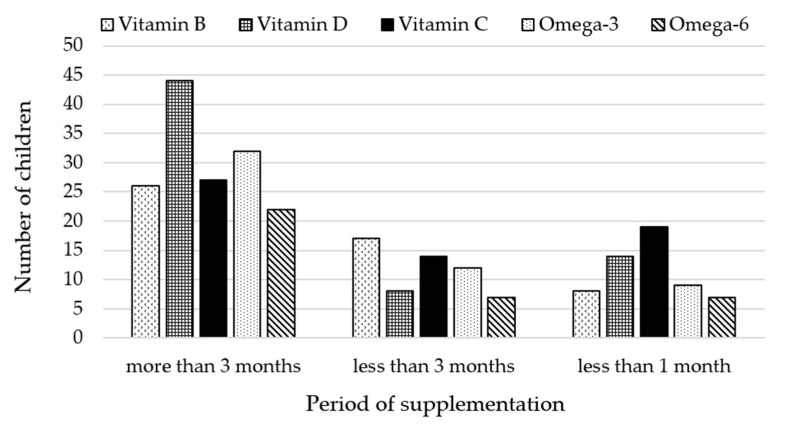
Number of children supplemented over a given period.

The duration of supplementation with a given supplement was different for each child. The children could be divided into the following three groups: supplemented for more than 3 months, less than 3 months and supplemented less than 1 month. Data on the consumed supplements and the duration of supplementation are presented in Figure 5. Additional graphs showing data on vitamin B and D intake with specification of vitamin type, doses used and duration of supplementation are presented in the Supplementary Materials (Figures S3 and S4).

### 4.2. Sample Preparation

Urine samples were thoroughly mixed and aliquoted to maintain homogeneity. Urine was transferred to 1.5 mL Eppendorf tubes and then stored at −20 °C until GC-MS analysis was performed. The sample preparation was performed using the method described by Zhang et al. [79] with simple modifications. For the analysis, the samples were thawed at ambient temperature and centrifuged at 18,000 rpm for 2 min. Using diethyl ether and ethyl acetate, the compounds were extracted. The supernatant was evaporated, and the remaining dry residue was derivatized using an 80 μL mixture of N, O-Bis(trimethylsilyl) trifluoroacetamide (BSTFA) and 1% trimethylchlorosilane (TMCS). The derivatization process was carried out in an ultrasonic bath for 10 min at 60 °C. All samples were centrifuged, transferred to glass chromatographic vials and then subjected to the GC/MS analysis. To calculate the organic acid concentrations in the urine samples, calibration curves based on the values of the peaks of standard HVA and VMA acid solutions were established. The linear relationship for HVA ranges from 1 μg/mL to 400 μg/mL and for VMA from 10 μg/mL to 150 μg/mL with a coefficient of determination (*r*^2^) of >0.99 for both. The LOD was determined as the concentration equivalent to an S/N of 3. The precision (CV) of measurements is 5.16% for HVA and 5.22% for VMA. In each child, HVA and VMA concentrations were normalized to the creatinine concentrations to account for changes in the glomerular filtration rate. The results were calculated as a ratio of the analyte of interest and urinary creatinine concentration in the unit μg/mg creatinine (μg/mg C).

### 4.3. Solvents and Derivatizing Agents for GC-MS Analysis

Standard substances of HVA and VMA acids (>99%), N,O-bis(trimethylsilyl)trifluoroacetamide (BSTFA) and trimethylchlorosilane (TMCS) were obtained from Sigma-Aldrich Inc. (St. Louis, MO, USA). HPLC solvent grade ethyl acetate, diethyl ether and chloroform were purchased from Merck (Darmstadt, Germany), and analytical-grade hydrochloric acid and sodium chloride were obtained from POCh (Gliwice, Poland).

### 4.4. Urinary Creatinine Determination

The level of HVA and VMA in urine was standardized by conversion to the creatinine level. The creatinine level was investigated using the high-performance liquid chromatography method reported elsewhere [37,48].

### 4.5. Analytical Methods

GC-MS analysis was performed with an Agilent 6890N Network GC system and a 5973 Network Mass Selective equipped with a capillary column (J&W Ultra Inert HP-5ms; Agilent Technology, Santa Clara, CA, USA; 30 m × 0.25 mm internal diameter; film thickness, 0.25 μm). Onto the analytical column, 1 μL of supernatant was injected. Helium with a constant flow of 0.9 mL/min was used as the carrier gas. The injection temperature was 250 °C. Initially, the column was heated to 75 °C and this temperature was maintained for 5 min. Then the column temperature was increased by 15 °C/min to the temperature of 280 °C. The mass spectrometer (MS) was operated in the electron impact (EI) mode at 70 eV ionization energy. The temperature of the MS quadrupole was set to 150 °C and the temperature of the ion source was set to 230 °C. Masses were acquired from *m*/*z* 50–550. Mass Hunter Workstation Software was used for qualitative and quantitative analysis of compounds. The full mass spectra were used for the structural identification of analytes and attribution of proper retention time to each compound under analysis. Electron impact mass spectra data for the trimethylsilyl derivatives of HVA and VMA were proved by the NIST mass spectra library. The mass spectra of the metabolites under study coincided with the corresponding mass spectra from the NIST spectral mass library. Characteristic *m*/*z* values of trimethylsilyl derivatives of HVA and VMA were chosen based on available literature data [80,81,82,83] and our previous studies [43]. Mass spectra for HVA and VMA in urine samples, spectra from the spectra library NIST.08 and a comparison of collected sample mass spectra and the corresponding library reference spectra for determined metabolites are presented in the Supplementary Materials (Figures S5 and S6). Qualifying ions, monitored in the specific selected-ion monitoring SIM mode, were *m*/*z* 179, 209, 326 for HVA; *m*/*z* 267, 297 for VMA. The underlined ions were used for quantification. The results of the analyses are presented in μmol/mmol creatinine.

### 4.6. Statistical Analysis

The results are presented as the mean ± standard deviation (SD). Outliers were identified as values outside the mean ± 3SD interval and excluded from statistical tests. Variables were checked for normality by the Shapiro–Wilk’s test. Nonparametric Mann–Whitney U test was used to compare the concentration of carboxylic acids between the groups. All comparisons were two-sided with a *p*-value of less than 0.05 used to indicate statistical significance. Subsequently, correlation analysis was carried out to discover possible relations among the variables. Statistical analysis was performed using Statistica 9.0 software (StatSoft, Poland STATISTICA, version 9.0, Quest Software, Aliso Viejo, CA, USA).

## 5. Conclusions

The study shows that the levels of HVA and VMA in the urine of children with autism were higher compared to the reference values from typically developing children, which may be related to an abnormal functional imbalance of the dopamine system observed in ASD. Supplementation with omega-3 and -6 fatty acids influences the levels of HVA and VMA in the study group. Supplementation with omega-3 fatty acids increased the level of VMA and supplementation with vitamin B lowered the level of HVA. In some children, a reduction in the severity of autistic symptoms was also observed, e.g., children responded better to their own name or had a better reaction to changes. This information suggests that children with ASD may have alteration in transmitter metabolism, abnormal functional imbalance of the dopamine system and demonstrate dysregulations in micronutrient, vitamin and other nutrients availability due to the nutrition problems and metabolic imbalance. Our results provide insights into the molecular processes altered in ASD. A comprehensive nutritional and dietary intervention can be an effective tool in improving nutritional status, metabolic and neurotransmitter status, and other symptoms in most individuals with ASD. More research is needed to further investigate the effects of supplementation on children’s behaviour, physical symptoms and levels of HVA and VMA among children with ASD.

## Figures and Tables

**Table 1:** Stratification of tested population (mean ± SD).

**Name of Compound**	**Unit**	Supplementation	Mean ± SD	Median	Min	Max	LQ	UQ	SE	*p*-Value *	References Values **
HVA	μmol/mmol creatinine	yes	26.62 ± 12.54	24.27	8.10	85.51	18.61	32.10	1.25	**7.99 × 10^−8^**	9.74 ± 3.99
		no	30.04 ± 13.32	27.58	11.35	68.42	20.38	38.01	2.66		
VMA		yes	12.92 ± 8.32	11.19	0.33	56.82	7.19	16.76	0.82	**2.70 × 10^−9^**	7.76 ± 2.94
		no	11.25 ± 4.15	11.87	0.90	19.18	8.00	14.27	0.83		

SD—standard deviation, LQ—lower quartile, UQ—upper quartile, SE—standard error, *—*p* values calculated by a Shapiro–Wilk test, **—reference range available online at The Human Metabolome Database.

**Table 2:** Comparison of determined HVA and VMA levels with consumed supplements and Mann–Whitney U test value.

**Name of Compound**	Unit	Mean ± SD	*p*-Value					
			Vitamin B	Vitamin C	Vitamin D	Omega-3	Omega-6	Probiotic
HVA	μmol/mmol creatinine	27.31 ± 12.72	**0.0164**	0.8679	0.9823	0.4189	**0.0015**	0.1376
VMA		12.59 ± 7.70	0.3620	0.3876	0.1638	0.8189	**0.0025**	0.3962

**Table 3:** Characteristics of studied population.

Number of Analyzed Samples	N = 129	Boys = 120
		Girls = 9
**Age Ranges**	3–18 years of age	
**number of underweight** **children**	13	
**number of normal weight children**	93	
**number of overweight** **children**	22	
**number of children with obesity**	1	

**Table 4:** Characteristics of studied population regarding supplementation.

		Supplementation		Dose/Composition	
		Yes	No	Min.	Max.
**Omega-3**	**EPA**	60	69	92 mg	800 mg
	**DHA**	60	69	26 mg	400 mg
**Omega-6**	**GLA**	19	69	10.5 mg	48.2 mg
	**LA**	7	69	348 mg	2000 mg
**Vitamin C**		58	71	50 mg	2200 mg
**Vitamin D_3_**		66	63	200 IU (5 μg)	2000 IU (50 μg)
**Vitamins B**	**B1**	6	103	50 mg	100 mg
	**B3**	6	97	8 mg	16 mg
	**B6**	26	97	2.5 μg	200 mg
	**B12**	17	97	1 μg	10 mg
**Probiotics**		50	79	Contains no less than 2.5 billion *Lactobacillus*, *Bifidobacterium*, *Streptococcus* and *Lactococcus* bacteria.	

EP—eicosapentaenoic acid; DHA—docosahexaenoic acid; GLA—γ-linolenic acid; LA—linoleic acid.

## Data Availability

The data presented in this study are available on request from the corresponding author. The data are not publicly available due to restrictions on privacy.

## Supplementary Materials

The following supporting information can be downloaded at: https://www.mdpi.com/article/10.3390/metabo12050423/s1, Figure S1: Box and Whisker plots for the compounds determined in vitamins B supplementation-categorized children with autism group. In these box plots, medians inside the 25–75% interquartile range (IQR) are presented; Figure S2: Box and Whisker plots for (a) the level of HVA determined in gender-categorized children with autism group; (b) the level of VMA determined in gender-categorized children with autism group. In these box plots, medians inside the 25–75% interquartile range (IQR) are presented; Figure S3: Number of vitamins B supplemented children over a given period; Figure S4: Number of children vitamin D supplemented considering the dose and duration of vitamin intake; Figure S5: EI mass spectrum of HVA-2TMS; A—mass spectrum of HVA-2TMS from a urine sample; B—mass spectrum of HVA-2TMS from the NIST mass spectra library; C—comparison of collected sample mass spectra and the corresponding library reference spectra for HVA-2TMS; Figure S6: EI mass spectrum of VMA-3TMS; A—mass spectrum of VMA-3TMS from a urine sample; B—mass spectrum of VMA-3TMS from the NIST mass spectra library; C—comparison of collected sample mass spectra and the corresponding library reference spectra for VMA-3TMS; Table S1: Supplementation table of observed HAV and VMA concentrations and creatinine in the urine of children with ASD; Table S2: Comparison of determined HVA and VMA levels with supplementation of vitamins B; Table S3: The determined correlation coefficients are significant with *p* < 0.05. Statistically significant correlations are highlighted. Spearman’s rank-order correlation test results.
