# Supplementary material for: Effect of Supplementation on Levels of Homovanillic and Vanillylmandelic Acids in Children with Autism Spectrum Disorders
Source: Metabolites. 2022 May 9;12(5):423. doi: 10.3390/metabo12050423 (PMC9145809; doi:10.3390/metabo12050423)
Supplement: Supplementary file 1 [file metabolites-12-00423-s001.zip › metabolites-1711582-supplementary.pdf]

Supplementary Material

Figure S1. Box and Whisker plots for the compounds determined in vitamins B supplementation-categorized autistic children group. In these box plots, medians inside the 25%–75% interquartile range (IQR) are presented.

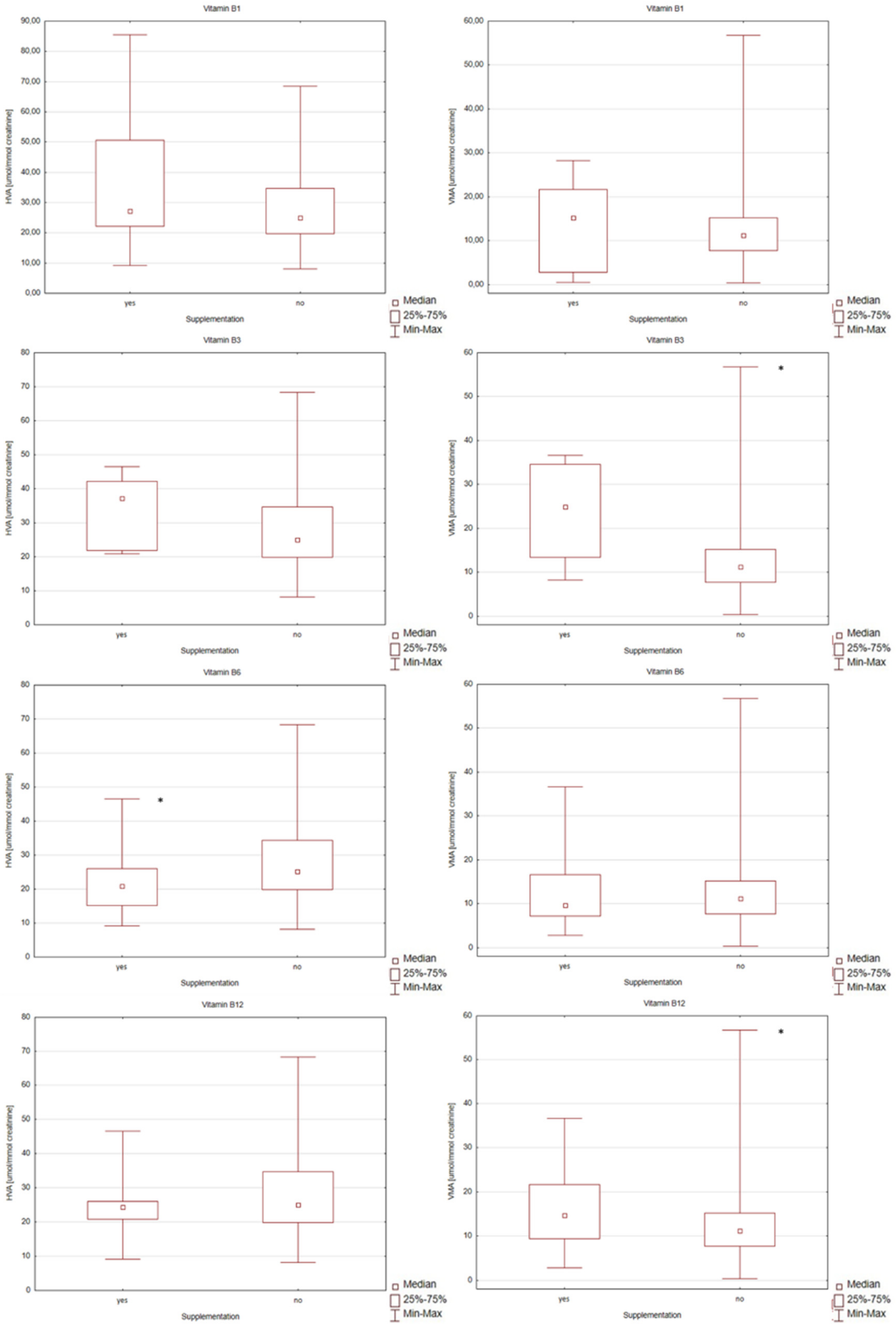

Figure S2. Box and Whisker plots for: (a) the level of HVA determined in gender-categorized autistic children group; (b) the level of VMA determined in gender-categorized autistic children group. In these box plots, medians inside the 25–75% interquartile range (IQR) are presented.

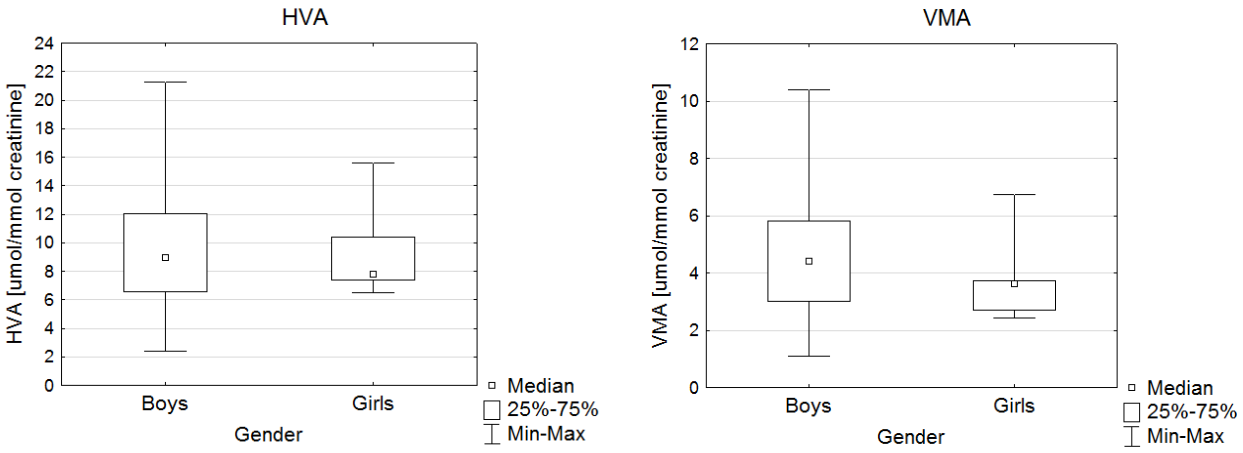

(a) **(b)**

Figure S3. Number of children vitamins B supplemented over a given period.

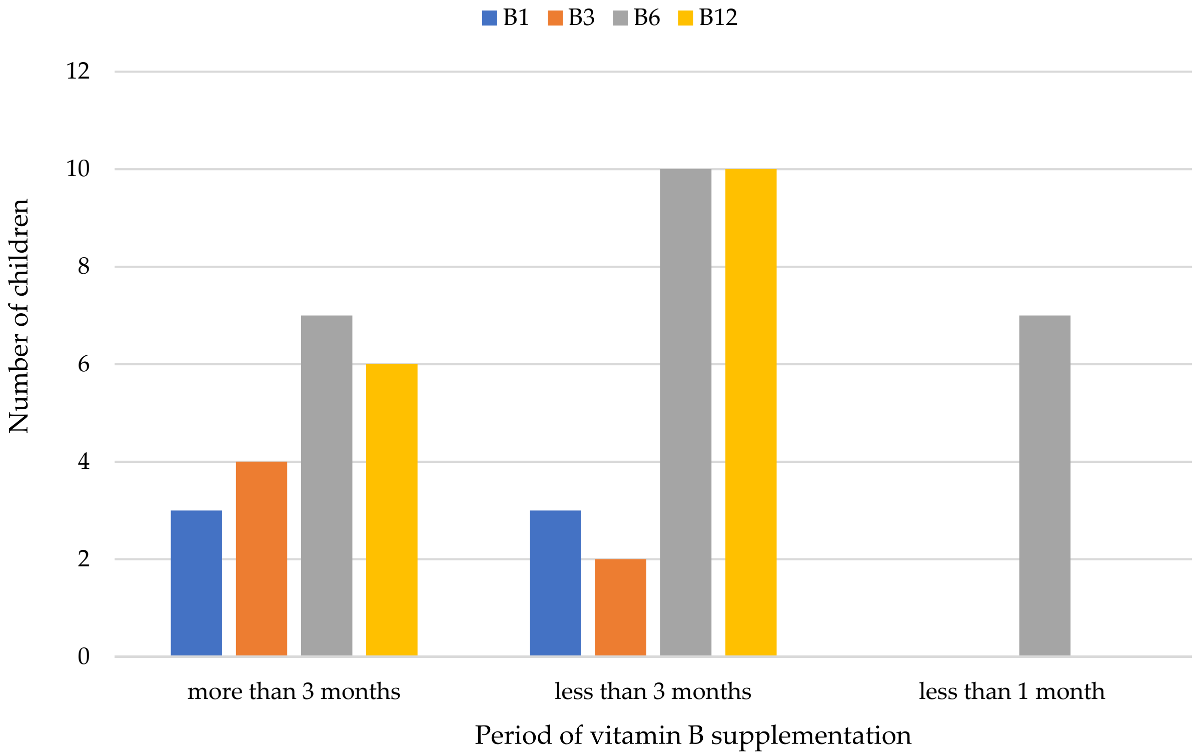

Figure S4. Number of children vitamin D supplemented considering the dose and duration of vitamin intake.

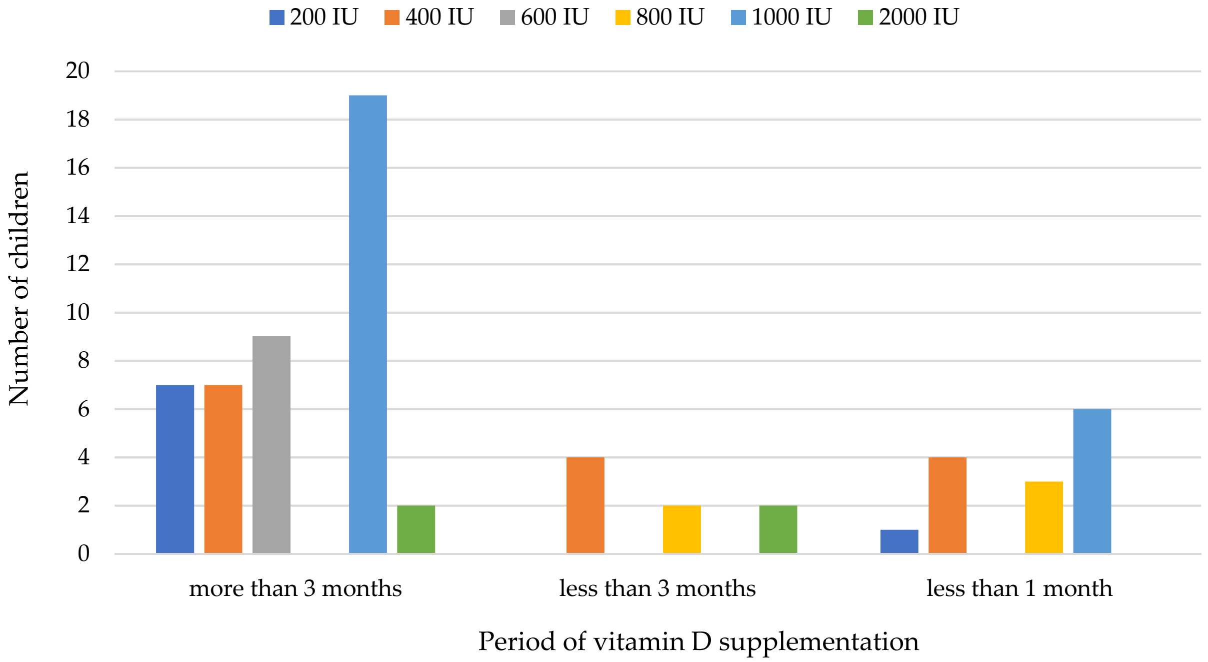

Figure S5. EI mass spectrum of HVA-2TMS; A - mass spectrum of HVA-2TMS from a urine sample; B - mass spectrum of HVA-2TMS from the NIST mass spectra library; C - comparison of collected sample mass spectra and the corresponding library reference spectra for HVA-2TMS.

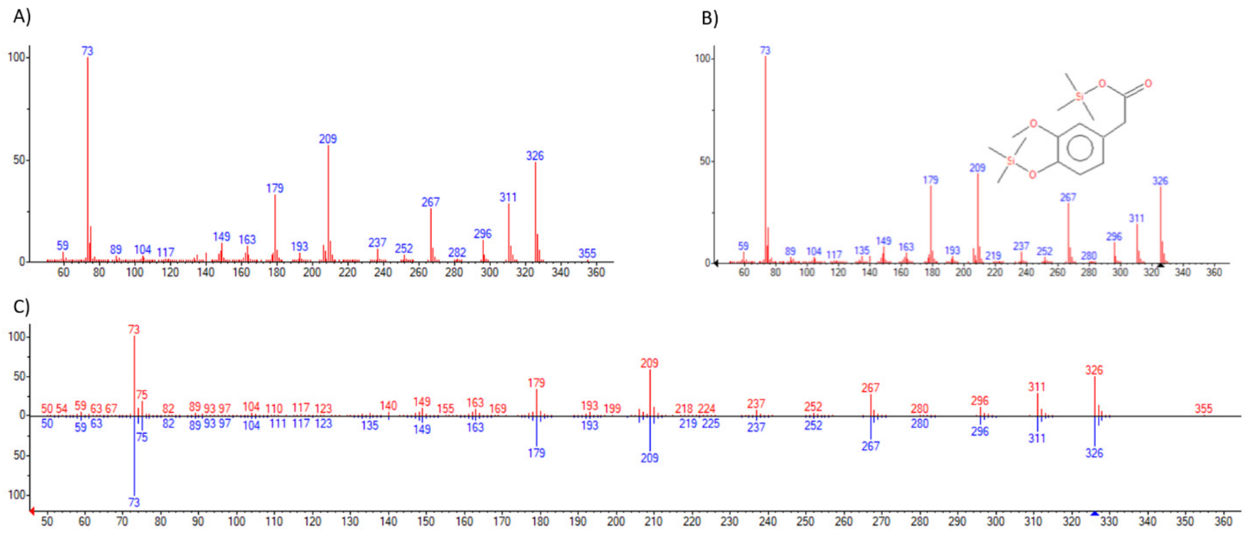

Figure S6. EI mass spectrum of VMA-3TMS; A - mass spectrum of VMA-3TMS from a urine sample; B - mass spectrum of VMA-3TMS from the NIST mass spectra library; C - comparison of collected sample mass spectra and the corresponding library reference spectra for VMA-3TMS.

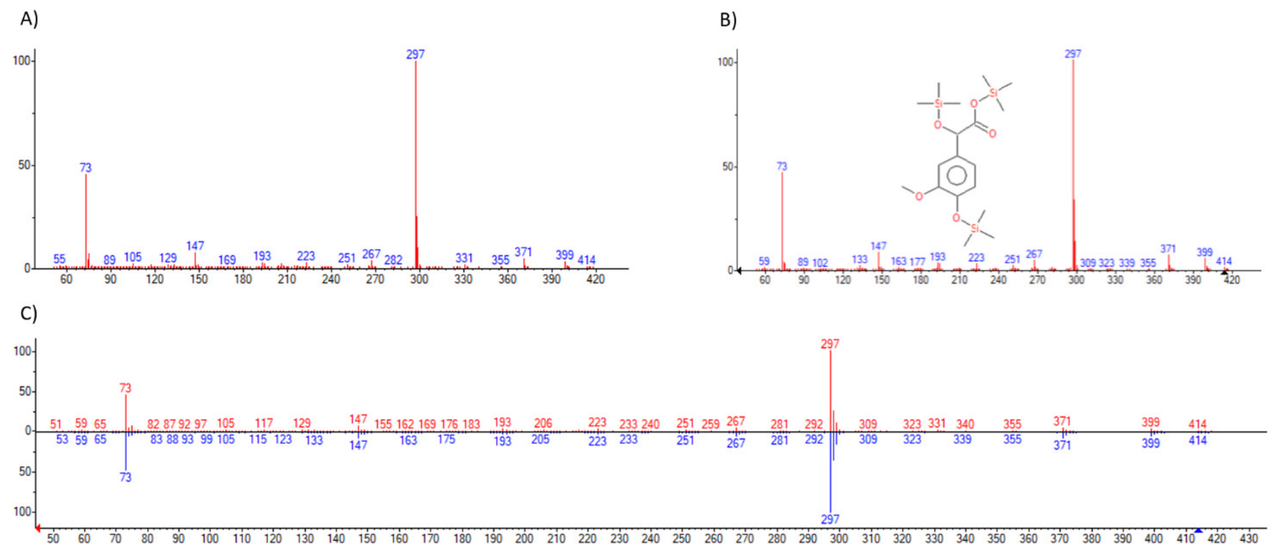

Table S1. Supplementation table of observed HVA and VMA concentrations and creatinine in urine of ASD children.

**No.**	**Concentration**			**Vitamin C**	**Vitamin B**	**Vitamin D3**	**Fatty acids**		**Probiotics**
	**HVA [µmol/mmol creatinine]**	**VMA [µmol/mmol creatinine]**	**Creatinine [µg/ml]**				**omega-3**	**omega-6**	
**1**	50.72	28.19	719.49	1	1	0	0	1	0
**2**	28.24	0.47	765.21	1	1	0	0	1	0
**3**	85.51	21.01	1668.07	1	1	0	0	1	0
**4**	34.88	9.75	688.07	1	1	0	0	1	1
**5**		12.05	808.07	1	1	0	0	1	1
**6**	18.99	7.76	1225.21	0	0	0	0	0	0
**7**	40.24	13.77	299.49	0	0	0	0	0	0
**8**	38.01	13.73	1193.78	0	0	0	0	0	0
**9**	33.63	15.54	636.64	0	0	0	0	0	0
**10**	25.40	17.99	1019.49	1	1	1	1	1	0
**11**	25.14	16.44	1085.21	1	1	1	1	1	0
**12**	18.97	14.45	890.92	1	1	1	1	1	0
**13**	42.76	12.85	139.49	0	0	0	0	0	0
**14**	29.90	16.78	342.35	0	0	0	0	0	0
**15**	68.42	19.18	82.35	0	0	0	0	0	0
**16**	52.42	0.90	399.49	0	0	0	0	0	0
**17**	27.97	15.57	825.21	0	0	0	0	0	0
**18**	23.01	12.97	713.78	0	0	0	0	0	0
**19**	43.76	24.41	382.35	1	1	1	0	0	1
**20**	15.24	7.15	616.64	1	1	1	1	0	1

**21**	19.85	16.58	1065.21	1	1	1	1	0	1
**22**	9.10	2.78	696.64	0	1	0	0	0	0
**23**	25.99	21.59	542.35	0	1	0	0	0	0
**24**	22.05	9.42	616.64	0	1	0	0	0	0
**25**	24.39	17.78	836.64	0	0	0	0	0	1
**26**	28.74	17.65	1508.07	0	0	0	0	0	1
**27**	32.45	18.34	570.92	1	0	1	0	0	0
**28**	26.98	13.80	325.21	1	0	1	0	0	0
**29**	27.39	13.52	496.64	1	0	1	0	0	0
**30**	50.77	20.20	408.07	1	0	1	0	0	0
**31**	34.32	13.76	402.35	1	0	1	0	0	0
**32**	36.77	23.97	299.49	1	0	1	0	0	0
**33**	23.16	8.98	699.49	0	0	1	1	0	0
**34**		22.03	905.21	0	0	1	0	0	0
**35**		0.33	1093.78	0	0	1	0	0	0
**36**	42.77	11.59	825.21	0	0	1	0	0	0
**37**	34.69	16.01	622.35	0	0	1	0	0	0
**38**	23.24	56.82	870.92	1	0	0	1	0	1
**39**	16.40	0.81	445.21	1	0	0	1	0	1
**40**	63.52	24.72	516.64	1	0	0	1	0	1
**41**	37.08	10.23	453.78	1	0	0	1	0	1
**42**	16.64	8.98	733.78	0	1	0	0	0	0
**43**	11.35	8.00	1679.49	0	0	0	0	0	0
**44**	15.90	5.76	796.64	1	0	0	0	0	1
**45**	20.77	8.25	442.35	1	1	1	1	0	0
**46**	21.88	13.34	1088.07	1	1	1	1	0	0
**47**	9.87	3.22	873.78	1	1	1	0	0	0
**48**	16.94	6.82	482.35	1	1	1	0	0	0
**49**	21.44	9.36	545.21	0	0	1	0	0	0
**50**	40.44	15.26	213.78	0	0	1	0	0	0
**51**	15.05	9.18	1268.07	1	0	1	1	0	1
**52**	24.90	14.03	1148.07	1	0	1	1	0	1
**53**	24.97	15.17	1248.07	1	0	1	1	0	1
**54**	16.83	13.08	842.35	0	0	0	1	0	0
**55**	26.30	11.13	496.64	0	0	0	1	0	0
**56**	20.13	9.99	1148.07	0	0	0	1	0	0
**57**	18.49	11.42	605.21	0	0	1	0	0	0
**58**		0.59	499.49	1	0	1	0	0	1
**59**	35.11	16.93	919.49	1	0	1	0	0	1
**60**	21.49	7.87	988.07	1	0	1	0	0	1
**61**	11.13	4.40	768.07	0	0	0	0	0	1
**62**	20.35	7.04	805.21	0	0	0	0	0	1
**63**	31.62	12.19	1216.64	0	0	0	0	0	1
**64**	20.86	9.06	645.21	1	1	1	1	0	1
**65**	15.07	13.55	1699.49	1	1	1	1	0	1
**66**	10.03	5.31	622.35	1	1	1	1	0	1
**67**	24.53	14.67	588.07	1	1	1	1	0	1
**68**	24.41	15.80	445.21	1	1	1	1	0	1

**69**	19.85	8.89	550.92	0	0	1	1	0	0
**70**	26.55	17.59	559.49	0	0	1	1	0	0
**71**	17.55	6.22	299.49	0	0	1	1	0	0
**72**	21.29	8.56	388.07	1	0	0	0	0	0
**73**	18.15	6.28	756.64	1	0	0	0	0	0
**74**	14.98	5.41	913.78	1	0	0	0	0	0
**75**	11.83	4.49	816.64	1	0	0	0	0	0
**76**	8.10	3.67	1096.64	1	0	0	0	0	0
**77**	11.09	5.50	510.92	1	0	0	0	0	0
**78**	29.96	11.48	759.49	0	0	1	1	0	0
**79**	28.98	11.42	593.78	0	0	1	1	0	0
**80**	22.88	11.07	228.07	0	0	1	1	0	0
**81**	22.30	10.10	416.64	0	0	0	0	0	0
**82**	21.76	6.84	553.78	0	0	0	0	0	0
**83**	20.38	9.33	753.78	0	0	0	0	0	0
**84**	27.58	9.70	962.35	0	0	0	0	0	0
**85**	13.00	6.25	705.21	0	0	0	0	0	0
**86**	19.77	14.34	608.07	0	0	0	0	0	0
**87**	35.02	11.03	330.92	0	0	1	1	1	0
**88**	50.39	19.29	490.92	0	0	1	1	1	0
**89**	37.44	21.85	353.78	0	0	1	1	1	0
**90**	24.16	6.64	1025.21	1	0	1	1	0	1
**91**	18.79	6.39	370.92	1	0	1	1	0	1
**92**	28.88	9.43	413.78	1	0	1	1	0	1
**93**	25.10	29.07	310.92	1	0	1	1	0	1
**94**	25.22	11.22	268.07	1	0	1	1	0	1
**95**	31.75	11.17	848.07	1	0	1	1	0	1
**96**	18.72	6.97	868.07	0	0	1	0	0	1
**97**	13.59	6.44	530.92	0	0	1	0	0	1
**98**	19.10	6.30	676.64	0	0	1	0	0	0
**99**	30.27	12.59	1379.49	1	0	1	1	1	1
**100**	21.53	10.49	376.64	1	0	1	1	1	1
**101**	45.67	15.20	668.07	0	0	0	0	0	0
**102**	40.07	13.28	616.64	0	0	0	0	0	0
**103**	34.64	11.87	796.64	0	0	0	0	0	0
**104**	20.88	7.69	830.92	0	0	1	1	1	0
**105**	27.02	18.07	636.64	0	0	1	1	1	0
**106**	30.04	13.04	593.78	0	0	1	1	1	0
**107**	29.50	13.13	668.07	1	0	0	0	0	1
**108**	20.39	8.64	419.49	1	0	0	0	0	1
**109**	21.84	10.48	1448.07	1	0	0	0	0	1
**110**	17.79	8.19	1039.49	0	0	0	1	0	1
**111**	56.42	21.13	599.49	0	0	0	1	0	1
**112**	43.23	19.34	230.92	0	0	0	1	0	1
**113**	49.65	20.32	405.21	0	0	0	1	0	1
**114**	19.88	5.91	576.64	0	0	0	0	0	1
**115**	16.91	5.42	650.92	0	0	0	0	0	1
**116**	36.14	16.50	1308.07	0	0	0	0	0	1

**117**	19.60	10.23	1216.64	0	0	0	0	0	0
**118**	18.87	6.50	1036.64	0	0	0	0	0	0
**119**	20.99	7.20	405.21	0	0	0	0	0	0
**120**	46.54	34.54	828.07	1	1	1	0	1	0
**121**	39.36	36.57	410.92	1	1	1	0	1	0
**122**	42.22	25.70	408.07	1	1	1	0	1	0
**123**	35.12	24.00	216.64	1	1	1	0	1	0
**124**	20.05	7.24	576.64	0	1	1	1	0	1
**125**	15.25	8.71	568.07	0	1	1	1	0	1
**126**	12.54	5.96	493.78	0	1	1	1	0	1
**127**	17.69	7.93	465.21	0	1	1	1	0	1
**128**	22.33	9.10	168.07	0	0	0	0	0	0
**129**	37.35	14.27	1430.30	0	0	0	0	0	0

Table S2. Comparison of determined HVA and VMA levels with supplementation of vitamins B.

**Name of the compound**	***p*-value**			
	**Vitamin B1**	**Vitamin B3**	**Vitamin B6**	**Vitamin B12**
HVA	0.4997	0.1120	**0.0499**	0.5861
VMA	0.7091	**0.0106**	0.8356	**0.0371**

Table S3. Determined correlation coefficients are significant with $p < 0.05$. Statistically significant correlations are highlighted. Spearman's rank-order correlation test results.

	Age	Gender	BMI	HVA	VMA	Vitamin C	Vitamin D	Probiotics	Omega -3 and -6 fatty acids	Vitamins B
Age	1,00	0,12	0,02	-0,53	-0,33	-0,20	-0,05	-0,15	-0,08	-0,24
Gender	0,12	1,00	-0,14	-0,10	-0,13	0,07	-0,28	-0,16	0,26	0,16
BMI	0,02	-0,14	1,00	0,12	0,11	0,25	0,21	-0,06	-0,14	0,01
HVA	-0,53	-0,10	0,12	1,00	0,74	0,03	0,00	0,13	-0,10	0,11
VMA	-0,33	-0,13	0,11	0,74	1,00	-0,07	0,12	0,08	-0,21	-0,08
Vitamin C	-0,20	0,07	0,25	0,03	-0,07	1,00	-0,27	0,29	0,25	0,37
Vitamin D	-0,05	-0,28	0,21	0,00	0,12	-0,27	1,00	-0,08	-0,40	-0,24
Probiotics	-0,15	-0,16	-0,06	0,13	0,08	0,29	-0,08	1,00	0,27	0,06
Omega -3 and -6 fatty acids	-0,08	0,26	-0,14	-0,10	-0,21	0,25	-0,40	0,27	1,00	0,35
Vitamins B	-0,24	0,16	0,01	0,11	-0,08	0,37	-0,24	0,06	0,35	1,00
